# Impact of the first wave of the COVID-19 pandemic on healthcare use in osteoarthritis: A population register-based study in Sweden

**DOI:** 10.1016/j.ocarto.2022.100252

**Published:** 2022-03-04

**Authors:** Ali Kiadaliri, Karin Magnusson, Aleksandra Turkiewicz, Andrea Dell’Isola, Jos Runhaar, Sita Bierma-Zeinstra, Martin Englund

**Affiliations:** a Clinical Epidemiology Unit, Orthopedics, Department of Clinical Sciences Lund, Lund University, Lund, Sweden; b Centre for Economic Demography, Lund University, Lund, Sweden; c Norwegian Institute of Public Health, Cluster for Health Services Research, Oslo, Norway; d Department of General Practice, Erasmus MC University Medical Center Rotterdam, Rotterdam, the Netherlands; e Department of Orthopedics & Sports Medicine, Erasmus MC University Medical Center Rotterdam, Rotterdam, the Netherlands

**Keywords:** Covid-19, Healthcare use, Event-study design, Osteoarthritis, Sweden

## Abstract

**Objective:**

To investigate whether the first wave of the COVID-19 pandemic impacted healthcare consultations (HCC) and hospitalization among people with and without osteoarthritis (OA).

**Methods:**

Using register data, we included individuals aged ≥35 years residing in Skåne region, Sweden, during 2009–2019 with (n ​= ​123,523) and without (n ​= ​552,412) a diagnosis of OA during January 1, 2009–December 31, 2019. We collected bi-weekly individual data on HCC/hospitalization between January and May for years 2017–2020. Treating the year 2020 as intervention and 2017–2019 as control as well as dividing data to pre– (January–February) and post–pandemic (March–May), we applied event study design to measure the dynamic effects of the COVID-19 pandemic on HCC/hospitalization. We used fixed-effect Poisson regressions for estimation and subgroup analyses by sex, age, and comorbidity were conducted among OA patients.

**Results:**

The impact of the pandemic on healthcare use was evident from mid-March 2020 (34–45%/12–25% reductions in in-person HCC/hospitalization) among people with OA relative to 2017–2019. Smaller reductions were seen in those without OA with 25–34%/8–16% reductions in in-person HCC/hospitalization. On contrary, there were increases in remote HCC following the pandemic (5–25% and 11–31% in people with and without OA, respectively). Among persons with OA, there were variations in the pandemic's effects by sex, age and comorbidity.

**Conclusion:**

Despite no lockdown in Sweden there were substantial reductions in in-person healthcare use during the first wave of COVID-19 pandemic with greater reductions among people with than without OA.

## 1 Introduction

The COVID-19 pandemic and response strategies to slow its spread have had significant health, economic, and social impacts [1]. The pandemic has imposed major changes in delivery of healthcare services and many healthcare providers restricted the provision of elective and non-essential healthcare procedures [2,3]. These changes have led to substantial drops in in-person healthcare use (HCU) for different types of care in different countries [[4], [5], [6], [7]]. These delayed/cancelled HCU might have adverse effects on patient's current and future health. These effects can be more pronounced among those with higher need of healthcare including older adults with chronic conditions. One such subgroup comprises patients with osteoarthritis (OA) who generally use more healthcare than individuals without OA [[8], [9]]. However, little is known about the impact of the COVID-19 pandemic on HCU in OA and in comparison to the general population. Using high-quality individual-level longitudinal register data, we investigated the patterns of HCU before and during the pandemic among people with and without OA in Sweden where *no* formal lockdown was implemented during the first wave of the COVID-19.

## 2 Method

We used data from the Swedish Population Register, the Skåne Healthcare Register (SHR), and the Longitudinal Integration Database for Health Insurance and Labour Market Studies (LISA by Swedish acronym) covering the entire Skåne population, the southernmost region of Sweden with about 1.4 million inhabitants. The SHR is a regional legislative administrative healthcare database covering all healthcare consultations (public and private) in the Skåne region from 1998 onwards. The registers were linked using the unique personal identification number, which was replaced with an arbitrary code by the Swedish authorities to ensure the anonymity of the subjects.

### 2.1 Study cohorts

We identified people aged ≥35 years who were resident in Skåne on 31 December 2019 (n ​= ​777,503). To have reliable data on OA diagnosis, we excluded those who moved to the region after December 31, 2008 (n ​= ​101,568). We then divided the sample into two cohorts: the *OA cohort* as those with a principal OA diagnosis (ICD-10 codes M15–M19, n ​= ​123,523) registered at least once during 2009–2019, and the *reference cohort* as those with no OA diagnosis during this period (n ​= ​552,412).

### 2.2 Healthcare use

We studied four types of HCU: (i) all in-person healthcare consultations (regardless of the reason for visit), (ii) all remote (phone and mail) healthcare consultations registered as those replacing an in-person visit, (iii) all hospital admissions (regardless of the reason for admission), and (iv) in-person mental health consultations (ICD-10 codes F00–F99). Healthcare consultations were computed as the sum of primary care and secondary outpatient care visits. Counting healthcare consultations was based on the date of visit, healthcare centre, and type of care provider. Hospital admissions expanded over 2 or more periods were counted as one admission only for the period of admission. We only studied in-person mental health consultations due to large fraction of missing diagnoses for remote consultations.

### 2.3 Analysis

We used an event-study design, a generalization of difference-in-difference (DID) model, to capture the dynamic effects of the COVID-19 pandemic on HCU [10,11]. In doing so, we treated the year 2020 (the pandemic year) as the intervention group and years 2017–2019 as the control group. We selected March 2, 2020 as the date of the pandemic onset because the Swedish Public Health Agency began recommendations in response to the COVID-19 pandemic in March 2020 [2] and first new COVID-19 infections were identified in the Skåne region in early March 2020 [2]. We then divided our data into pre- and post-pandemic for both intervention and control groups. The main idea is that in the absence of COVID-19, the average HCU in 2020 and 2017–2019 would have followed parallel trends over time (parallel trend assumption). We divided our data into 14-day periods and measured HCU for four pre-pandemic (5/6 January to 1 March) and six post-pandemic (2 March to 25 May) periods. We estimated the following regression model:

$$HCU_{ipy}=\overset{-1}{\underset{k=-3}{\sum }}\beta_{k}1\left(p-5=k\right)+\overset{5}{\underset{k=0}{\sum }}\alpha_{k}1\left(p-5=k\right)+\theta_{i}+\gamma_{p}+\delta_{y}+\varepsilon_{ipy}$$

where HCU_ipy_ represents the outcome of interest for individual i, in period p of year y. θ_i_ is individual fixed effects capturing time-invariant confounding factors, γ_p_ controls for temporal trends in HCU within a year, and δ_y_ captures the overall trend in HCU over time. 1 (p-5 ​= ​k) is an indicator for the 14-day periods relative to the pandemic onset period in 2020 (p ​= ​5, March 2–16, 2020). We set 1 (p-5 ​= ​k) to zero for all observations during 2017–2019. We used the first period (p ​= ​1, 5/6 to 19/20 January) as the baseline. α_k_ (β_k_) compares the changes in the outcome of interest between post-pandemic (pre-pandemic) periods and the baseline period in 2020 with the same changes during 2017–2019. Since HCU is a count variable, we estimated this model using Poisson fixed effect regression with standard errors clustered at individual level.

We conducted separate analyses for the OA cohort, which was the cohort of primary interest, and the reference cohorts. Among the OA cohort, we implemented subgroup analyses by sex (males and females), age (35–49, 50–64, 65–79, and 80+), and Elixhauser comorbidity index (0,1–2, and 3+). To check the robustness of our findings, we conducted a placebo test by excluding the data from the year 2020 and treating the year 2019 as the pandemic year and 2017–2018 as the control years.

## 3 Results

**Fig. 1:**
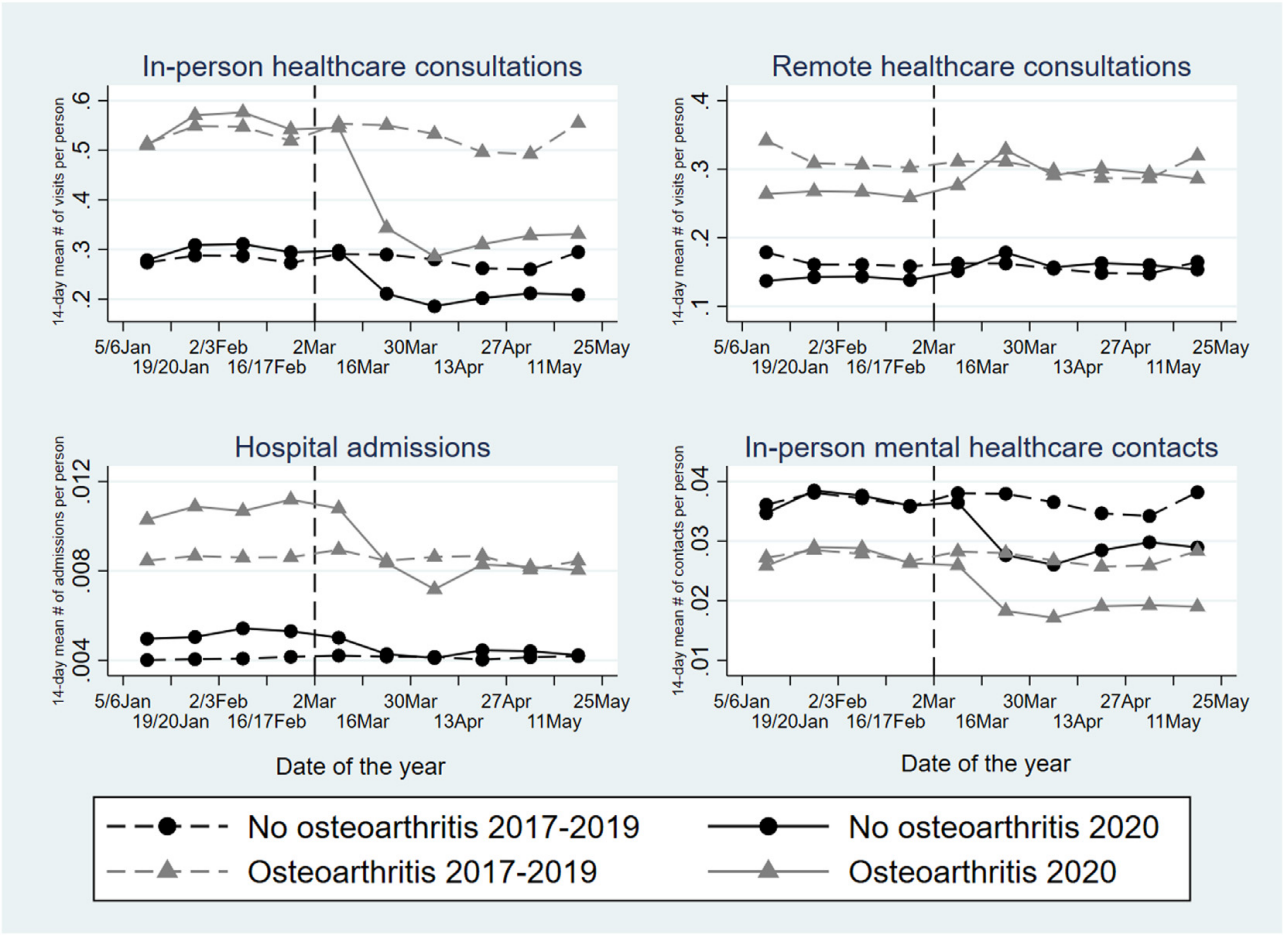
Temporal trends in healthcare use in persons with and without osteoarthritis in 2020 compared with 2017–2019. The vertical line divides the study period into pre- and post-pandemic.

Compared with the reference cohort, persons with OA were, on average, older (68 vs 55 years) with a higher proportion of females (61% vs 49%) among them (Table A1 in supplement). In addition, Elixhauser comorbidity index was higher among the OA cohort (median 2 vs 1). Exploring the patterns of HCU (Fig. 1) revealed that while the OA cohort had, on average, higher all healthcare consultations (in-person & remote) and hospital admissions than the reference cohort in all periods, the temporal trends were very similar. In addition, while people with OA had lower in-person mental healthcare contacts than the reference cohort, the temporal trends were comparable. Moreover, interruptions in HCU following the COVID-19 pandemic in 2020 is evident for almost all types of healthcare in both cohorts. It also can be seen that despite slight rises in in-person healthcare consultations from mid-April, it didn't return to the pre-pandemic level by the end of study period (May 2020).

**Fig. 2:**
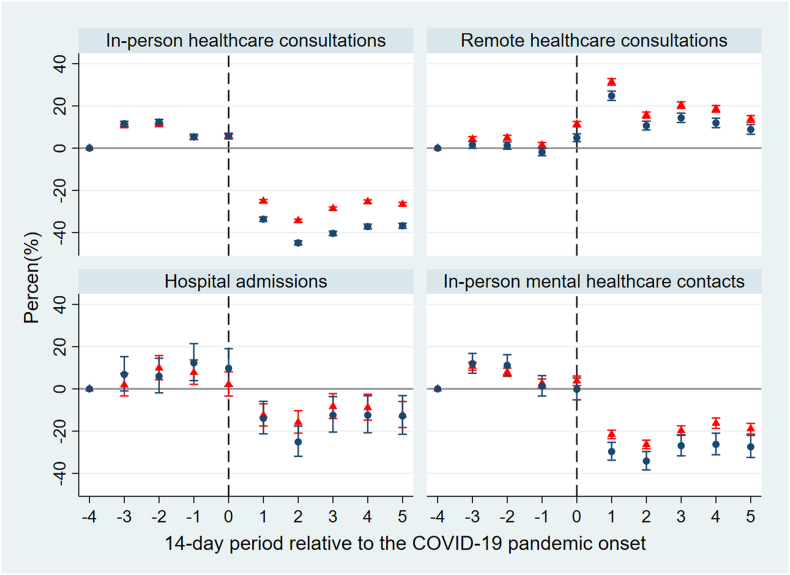
Effects of the COVID-19 pandemic on healthcare use in persons with (navy circles) and without (red triangles) osteoarthritis: estimates from event study. The vertical line divides the study period into pre- and post-pandemic.

The event study estimates showed that the COVID-19 pandemic was generally associated with reductions in in-person healthcare consultations and hospital admissions in both cohorts (Fig. 2) from the first period post-pandemic (mid-March) with more profound reductions in the OA cohort (34–45% vs 25–34% reductions in in-person healthcare consultations and 12–25% vs 8–16% reductions in hospital admissions). On the other hand, the pandemic was associated with rises in remote healthcare consultations from the pandemic week (early March) which lasted for the whole study period, with more profound increases among people without OA (5–25% in OA vs. 11–31% in the reference cohort). There were also declines in in-person mental health contacts following the pandemic with greater reductions among people with than without OA (26–34% vs.16–26%).

**Fig. A1:**
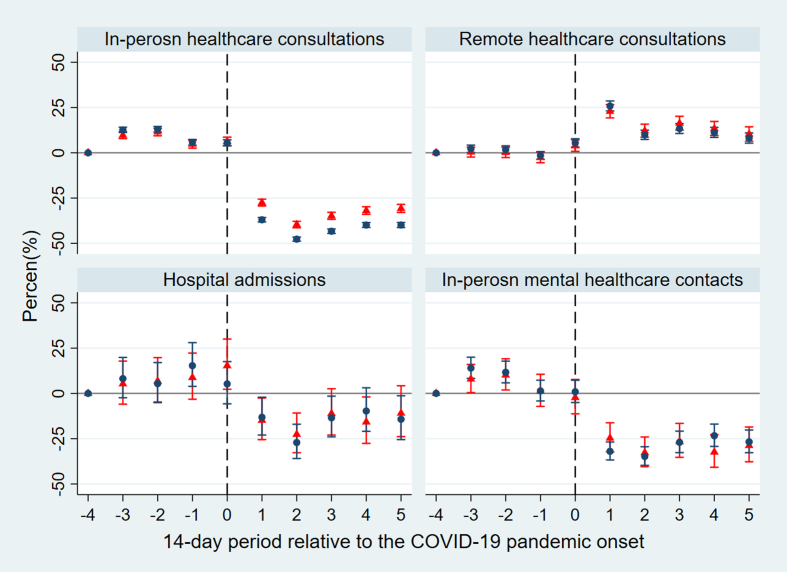
Effects of the COVID-19 pandemic on healthcare use in males (red triangles) and females (navy circles) with osteoarthritis: estimates from event study. The vertical line divides the study period into pre- and post-pandemic.

Our subgroup analyses in the OA cohort showed that the pandemic's effects generally tend to be larger among females, older people and those with comorbidity (Figures A1–A4 in supplement). Our placebo test did not suggest any interruptions in HCU in 2019 compared with 2017–2018 (Figure A5 in supplement).

## 4 Discussion

Using individual-level longitudinal data, we found that the COVID-19 pandemic in southern Sweden, despite no formal lockdown, was associated with substantial reductions in HCU that was more marked among people with than without OA. On the other hand, the pandemic was associated with increases in remote healthcare consultations which lasted for the whole study period. Among OA patients the pandemic's impact was generally greater for females, older people and those with more comorbidity.

Significant reductions in in-person HCU including hospital admissions and mental health consultations reported in this study is consistent with previous studies documenting steep reductions in different types of care following the COVID-19 pandemic compared to previous years [2,[4], [5], [6], [7],[10], [11], [12], [13]]. A recent systematic review [5] reported a median reduction of 42% in healthcare visits and 28% in hospital admissions following the pandemic which are slightly larger than the reductions estimated in our study. Smaller reductions observed in this study might be attributable to a lack of formal lockdown in Sweden. This suggests that while our estimates might not be directly transferable to countries with formal lockdown, these might provide a “lower bound” for the impact of COVID-19 on HCU in persons with OA. Moreover, this systematic review reported [5] greater reductions in healthcare consultations than hospital admissions which are in line with our findings.

A lack of formal lockdown in Sweden implies that the observed reductions in in-person HCU can be possibly attributed to people's reluctance to use healthcare due to their fear of the COVID–19 infection, recommendations of physical distancing and working from home by health authorities, and reductions of elective and non-essential care imposed by healthcare providers. Such voluntary actions should inform developing response strategies to mitigate the effects of potential future pandemics especially among those with higher need for healthcare services. In addition, reductions in HCU despite no formal lockdown highlight the importance of accounting for voluntary responses in estimating the causal impact of formal lockdown measures.

We observe increases in remote consultations in 2020 compared with previous years which lasts for the whole study period, with greater rises early on following the pandemic. This suggests that reductions in in-person consultations were partially offset by rises in remote consultations. The rise in remote consultations during the early phase of the pandemic followed by a reduction has also been reported in previous studies, even though the magnitude of the rises were smaller in our study [4,12,14]. This might partially be due the fact that video, phone visits and visits through mobile apps are not very frequent as compared to in-person visits. Furthermore, differences in study design, data sources and coding procedures might also contribute to these discrepancies. Further analyses are needed to explain smaller rises in remote consultations in this study. Indeed, if reductions in in-person HCU are not substituted by remote consultations, it can have detrimental effects on population health highlighting the need for further investigation.

The access to high-quality individual-level register data on healthcare contacts (publicly funded and given by both public and private providers) for the entire population of Skåne region is the main strength of this study. Furthermore, we used historical data to capture trends in the years leading up to 2020 which has been suggested to carry the lowest risk of bias for estimating the impact of the COVID-19 pandemic [5]. Nevertheless, several limitations of the current study should be acknowledged. No data on healthcare provided by the municipalities (e.g., nursing homes) was available in the register. Moreover, we lack data on certain types of remote consultations including video visits and mobile apps by certain private practitioners. We were unable to capture the OA severity. Potential variations in the pandemic's impact on HCU by OA severity (e.g. persons who underwent total joint replacement) and/or OA site should not be overlooked. Using physicians' diagnostic codes to identify patients with OA is susceptible to coding errors and misclassification, even though our data source had a high positive predictive value (88%) for knee OA diagnosis [15]. Given that Skåne was among the regions less impacted during the first wave of the COVID-19 pandemic, it is unclear if our results are generalizable to the whole of Sweden or other locations.

## 5 Conclusion

Compared with previous years, we observed significant reductions in in-person HCU following the COVID-19 pandemic in southern Sweden with more profound reductions among those with than without OA. Among people with OA, females, older individuals and those with more comorbidity experienced greater reductions in in-person healthcare consultations which might have adverse consequences for their health. Identifying the effect of pandemic on disease-specific HCU (e.g. infectious vs. non-infectious diseases) and the effect of formal lockdown on HCU in persons with OA are subjects for future research.

## Contributors

All authors contributed to study conception. AK contributed to study design and performed the statistical analysis, and drafted the manuscript. AT and ME participated in data acquisition. All authors contributed to the interpretation of the results, revising the manuscript critically for important intellectual content and approved the final version for submission.

## Credit author statement

**Ali Kiadaliri:** Conceptualization, Formal analysis, Visualization, Writing - Original Draft. **Karin Magnusson:** Conceptualization, Writing - Review & Editing. **Aleksandra Turkiewicz:** Conceptualization, Data Curation, Writing - Review & Editing. **Andrea Dell’Isola:** Conceptualization, Writing - Review & Editing. **Jos Runhaar:** Conceptualization, Writing - Review & Editing. **Sita Bierma-Zeinstra:** Conceptualization, Writing - Review & Editing, Funding acquisition. **Martin Englund:** Conceptualization, Funding acquisition, Supervision, Writing - Review & Editing.

## Funding

This study was supported by the Foundation for Research in Rheumatology (FOREUM), the 10.13039/501100006075Greta and Johan Kock Foundation, the 10.13039/501100004359Swedish Research Council, the Österlund Foundation, and Governmental Funding of Clinical Research within National Health Service (ALF).

## Patient consent for publication

Not required.

## Ethics approval

The study was approved by the Ethical Review Board, Sweden.

## Declaration of competing interest

AK acts as senior scientific advisor for Joint Academy, a platform providing evidence-based digital treatment for chronic joint pain. ME and SBZ has received personal fees from Pfizer outside the submitted work. All other authors have no conflict of interest to disclose.

## Figures and Tables

**Fig. A2:**
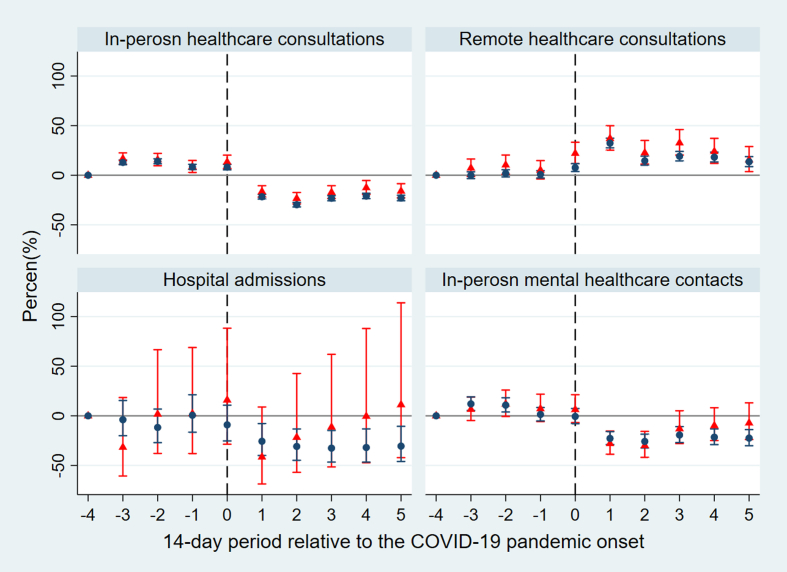
Effects of the COVID-19 pandemic on healthcare use in persons with osteoarthritis aged 35-49 (red triangles) and 50-64 (navy circles) years: estimates from event study. The vertical line divides the study period into pre- and post-pandemic.

**Fig. A3:**
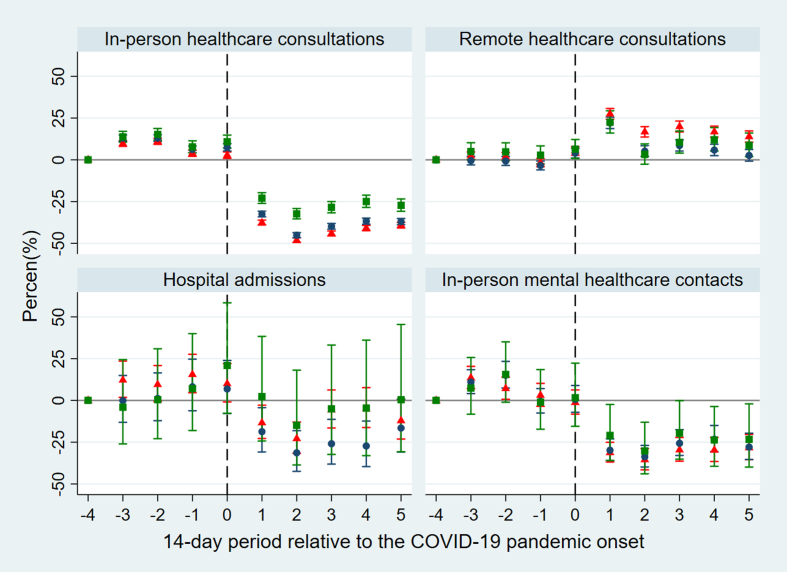
Effects of the COVID-19 pandemic on healthcare use in persons with osteoarthritis aged 65-79 (red triangles) and 80+ (navy circles) years: estimates from event study. The vertical line divides the study period into pre- and post-pandemic.

**Fig. A4:**
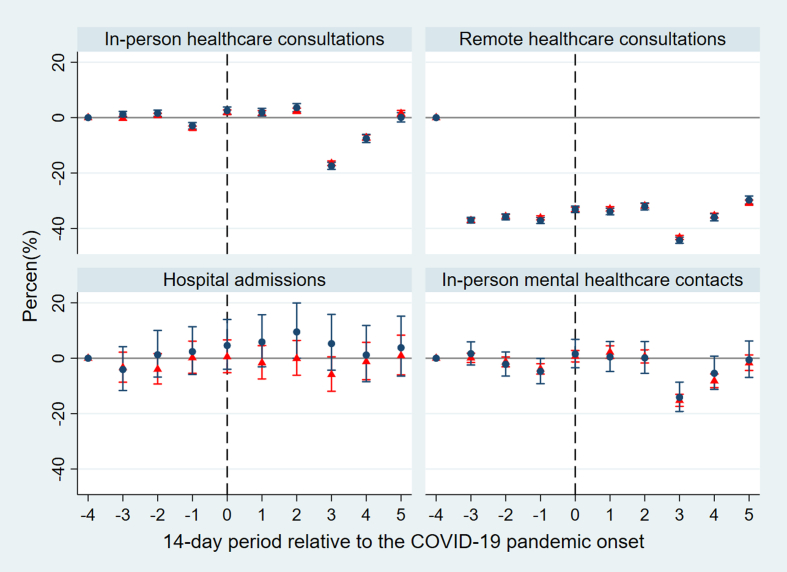
Effects of the COVID-19 pandemic on healthcare use in persons with osteoarthritis with no comorbidity (green squares), 1-2 (navy circles) and 3+ comorbidity (red triangles): estimates from event study. The vertical line divides the study period into pre- and post-pandemic.

**Fig. A5:**
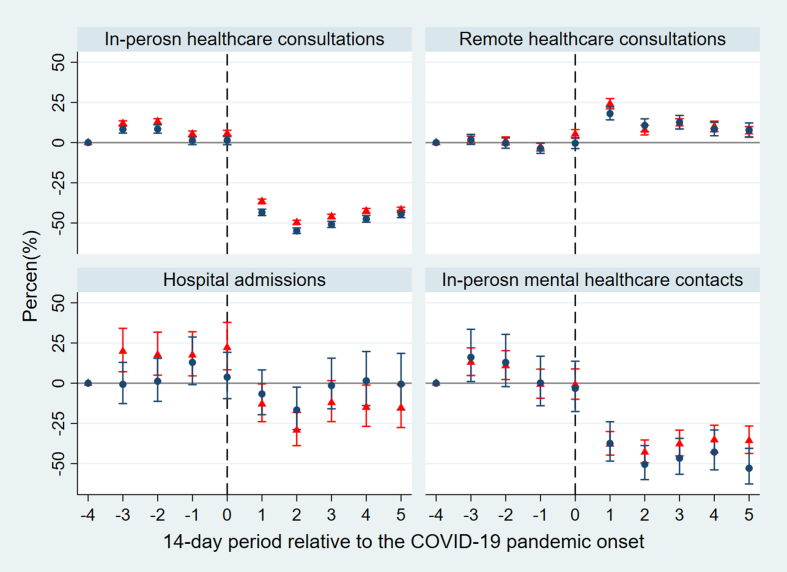
The results of placebo test in persons with (navy circles) and without (red triangles) osteoarthritis: estimates from event study. The vertical line divides the study period into (hypothetical) pre- and post-pandemic.
