# Supplementary material for: Impact of the first wave of the COVID-19 pandemic on healthcare use in osteoarthritis: A population register-based study in Sweden
Source: Osteoarthr Cartil Open. 2022 Mar 4;4(2):100252. doi: 10.1016/j.ocarto.2022.100252 (PMC8894697; doi:10.1016/j.ocarto.2022.100252)
Supplement: Multimedia component 1 [file mmc1.docx]

Table A1. Background characteristics of participants

| Variable | Osteoarthritis | Without osteoarthritis |
| --- | --- | --- |
| N | 123,523 | 552,412 |
| Females, % | 61.1 | 49.4 |
| Age (years), mean (SD) | 67.5 (12.0) | 55.2 (14.4) |
| Age (years), % |  |  |
| 35-49 | 5.5 | 35.3 |
| 50-64 | 27.8 | 33.4 |
| 65-79 | 45.6 | 23.5 |
| 80+ | 21.1 | 7.8 |
| Years of schooling, % |  |  |
| 0-9 | 26.1 | 17.6 |
| 10-12 | 44.8 | 43.0 |
| 13+ | 28.4 | 38.5 |
| Missing | 0.7 | 0.9 |
| Marital status, % |  |  |
| Not married | 12.1 | 24.8 |
| Previously married | 33.9 | 22.4 |
| Registered partner | 54.0 | 52.8 |
| Born in Sweden, % | 84.8 | 80.5 |
| Elixhauser comorbidity index ^a^, mean (SD) | 2.1 (1.9) | 1.1 (1.6) |
| Elixhauser comorbidity index ^a^, % |  |  |
| 0 | 22.8 | 48.7 |
| 1 | 23.2 | 22.4 |
| 2 | 19.5 | 12.7 |
| 3+ | 34.5 | 16.2 |

^a^ During 2016-2019
